# Gene Panel Tumor Testing in Ovarian Cancer Patients Significantly Increases the Yield of Clinically Actionable Germline Variants beyond *BRCA1*/*BRCA2*

**DOI:** 10.3390/cancers12102834

**Published:** 2020-09-30

**Authors:** Ana Barbosa, Pedro Pinto, Ana Peixoto, Joana Guerra, Carla Pinto, Catarina Santos, Manuela Pinheiro, Carla Escudeiro, Carla Bartosch, João Silva, Manuel R. Teixeira

**Affiliations:** 1 Cancer Genetics Group, IPO-Porto Research Center (CI-IPOP), Portuguese Oncology Institute of Porto (IPO Porto), 4200-072 Porto, Portugal; ana.barbosa@ipoporto.min-saude.pt (A.B.); pedro.pinto@ipoporto.min-saude.pt (P.P.); analuisamoura@ipoporto.min-saude.pt (A.P.); joana.goncalves.guerra@ipoporto.min-saude.pt (J.G.); carla.a.pinto@ipoporto.min-saude.pt (C.P.); catarinasantos@ipoporto.min-saude.pt (C.S.); manuelap@ipoporto.min-saude.pt (M.P.); carla.escudeiro@ipoporto.min-saude.pt (C.E.); joao.pinho.silva@ipoporto.min-saude.pt (J.S.); 2 Department of Genetics, Portuguese Oncology Institute of Porto (IPO Porto), 4200-072 Porto, Portugal; 3 Department of Pathology, Portuguese Oncology Institute of Porto (IPO Porto), 4200-072 Porto, Portugal; carla.bartosch@ipoporto.min-saude.pt; 4 Institute of Biomedical Sciences Abel Salazar (ICBAS), University of Porto, 4050-313 Porto, Portugal

**Keywords:** ovarian cancer, tumor testing, next generation sequencing, multi-gene panel, genetic predisposition, clinically actionable alterations

## Abstract

**Simple Summary:**

Germline and somatic variant testing of the *BRCA1* and *BRCA2* genes are important to predict treatment response to PARP inhibitors in ovarian cancer patients. However, germline variants in other genes besides *BRCA1* and *BRCA2* are associated with ovarian cancer predisposition, which would be missed by a genetic testing aimed only at treatment decision. We aimed to evaluate the yield of clinically actionable germline variants using next-generation sequencing of a customized panel of 10 genes for the analysis of pathology samples of ovarian carcinomas. We identified clinically actionable germline variants in a significantly higher proportion of ovarian cancer patients when compared with genetic testing focused only on *BRCA1* and *BRCA2*. This strategy increases the chance to make available genetic counseling, presymptomatic genetic testing, and gynecological cancer prophylaxis to female relatives who turn out to be healthy carriers of deleterious germline variants.

**Abstract:**

Since the approval of PARP inhibitors for the treatment of high-grade serous ovarian cancer, in addition to cancer risk assessment, *BRCA1* and *BRCA2* genetic testing also has therapeutic implications (germline and somatic variants) and should be offered to these patients at diagnosis, irrespective of family history. However, variants in other genes besides *BRCA1* and *BRCA2* are associated with ovarian cancer predisposition, which would be missed by a genetic testing aimed only at indication for PARP inhibitor treatment. In this study, we aimed to evaluate the yield of clinically actionable germline variants using next-generation sequencing of a customized panel of 10 genes for the analysis of formalin-fixed paraffin-embedded samples from 96 ovarian carcinomas, a strategy that allows the detection of both somatic and germline variants in a single test. In addition to 13.7% of deleterious germline *BRCA1*/*BRCA2* carriers, we identified 7.4% additional patients with pathogenic germline variants in other genes predisposing for ovarian cancer, namely *RAD51C*, *RAD51D*, and *MSH6*, representing 35% of all pathogenic germline variants. We conclude that the strategy of reflex gene-panel tumor testing enables the identification of clinically actionable germline variants in a significantly higher proportion of ovarian cancer patients, which may be valuable information in patients with advanced disease that have run out of approved therapeutic options. Furthermore, this approach increases the chance to make available genetic counseling, presymptomatic genetic testing, and gynecological cancer prophylaxis to female relatives who turn out to be healthy carriers of deleterious germline variants.

## 1. Introduction

Ovarian cancer (OC) is the third most common malignant disease among patients with gynecological neoplasia, being the eighth most common cancer and the eighth most common cause of cancer-related death in women [1]. The lack of specific symptoms and effective early detection strategies contributes to the high mortality rate, as most OC are diagnosed at an advanced stage [2]. Epithelial ovarian cancer (EOC) is recognized as a heterogeneous disease mainly represented by five histological subtypes (high-grade serous, low-grade serous, clear cell, endometrioid, and mucinous) that are characterized by distinct clinical and molecular features. Despite this knowledge, the initial standard treatment has typically been maximal effort cytoreductive surgery, with platinum-paclitaxel chemotherapy being the standard of care treatment [3,4]. Despite the usually good initial response, about 70–80% of patients will relapse within the subsequent three years and have limited treatment options thereafter [5,6].

The first poly(ADP-ribose) polymerase (PARP)-inhibitor (PARPi) received the approval from the European Medicines Agency (EMA) in 2014 for use as a maintenance therapy in relapsed high-grade serous ovarian cancer (HGSOC) patients with platinum-sensitivity and with *BRCA1/BRCA2* somatic or germline variants [7,8]. The same treatment was approved later for first-line maintenance therapy in BRCA mutated advanced EOC [9]. The mechanism underlying the efficacy of PARPi is synthetic lethality, which occurs in cells with deficiency in homologous recombination (HR) repair, where BRCA1/BRCA2 proteins play an essential role. When base excision repair (BER) is blocked by PARPi and thereby inhibits single strand break repair, the combined effect with HR results in accumulation of DNA aberrations and cell death [10]. Since PARPi approval, BRCA status became a relevant criterion to select patients who may benefit from this therapy or to identify the main responders to this class of drugs. Consequently, OC patients have been referred for *BRCA1* and *BRCA2* genetic testing (germline and somatic) to guide treatment decisions in addition to familial risk assessment regardless of family history of breast or OC [11]. This seems to be the best approach as some studies showed that more than 30% of women with germline variants had no family history of breast or OC [12].

Although most are sporadic, it has been reported that about 23% of OC are associated with hereditary predisposition [12], and a large proportion of these cases are due to inherited variants in *BRCA1* and *BRCA2* genes. Women with germline variants in these genes are at higher risk of developing OC, when compared with women in the general population, with the lifetime risk being 44% and 17% for *BRCA1* and *BRCA2*, respectively [13]. The use of next-generation sequencing (NGS) with multi-gene panels may allow the identification of other genes associated with inherited predisposition to OC [12]. Recent studies with blood samples showed that 18–24% of OC patients carry germline variants in genes associated with OC risk and, of these, 16–18% had germline variants in *BRCA1* or *BRCA2* genes and 4–6% had variants in other genes such as *BRIP1*, *RAD51C*, and *RAD51D* [12,14]. The identification of carriers of deleterious germline variants in OC predisposition genes allows the use of prophylactic surgery for a disease that currently has no effective screening allowing early diagnosis.

Many laboratories are now performing *BRCA1*/*BRCA2* testing in formalin-fixed paraffin-embedded (FFPE) tumor samples to detect germline and somatic variants to identify, in a single test, those patients with somatic and germline variants who are most likely to benefit from PARPi therapy. Using this strategy, there is a risk that patients without *BRCA1*/*BRCA2* variants are not evaluated for other genes predisposing to OC, thereby missing the opportunity for presymptomatic testing and prophylactic measures. Eventually, they might subsequently be referred for germline NGS testing of other genes, which increases the sequencing costs [11]. In this study, we designed a customized NGS gene panel that includes all known OC cancer predisposing genes for the detection of pathogenic germline variants in a single test using FFPE tumor samples. We show that this strategy significantly increases the yield of clinically actionable pathogenic germline variants in a single NGS test, which can subsequently be evaluated in a peripheral blood sample by Sanger sequencing for its eventual constitutional origin after genetic counseling.

## 2. Results

### 2.1. Variant Analysis

Of 96 FFPE OC samples, one (OC65) was excluded from further analysis because it did not pass quality filters. Among the ten genes included in the NGS panel (*BRCA1*, *BRCA2*, *BRIP1*, *MLH1*, *MSH2*, *MSH6*, *PMS2*, *RAD51C*, *RAD51D*, and *TP53*), a total of 3457 variants was annotated and classified among the remaining 95 tumor samples. After variant filtering 97 variants remained, of which 57 were classified as pathogenic/likely pathogenic in ClinVar. Among the variants not classified in ClinVar, two variants were nonsense, seven were frameshift variants, two were canonical +1 and −2 splice site variants, and one was an inframe variant, all of which were considered pathogenic since there is strong evidence of pathogenicity [15]. Additionally, there were 28 missense variants not classified, with unknown significance or conflicting interpretations of pathogenicity. The potential pathogenicity of these missense variants was evaluated through in silico prediction tools, and 19 of these variants were predicted to have a deleterious impact on the protein. We, therefore, retained 88 variants across seven genes, of which 69 (77.3%) were pathogenic and 19 (22.7%) were missense variants of uncertain significance (VUS). Of 95 tumor samples analyzed, variants in the genes under study were identified in 66 (69.5%).

### 2.2. Germline Variants

**Figure 1:**
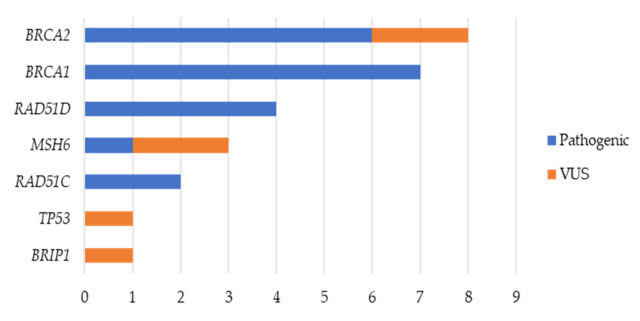
Spectrum of germline variants (pathogenic and variants of uncertain significance (VUS)).

After variant filtering, all variants of the genes of interest identified by NGS in the tumor of the 66 patients were tested in peripheral blood samples, and only those which were confirmed to be germline are included in this report. Of the 88 variants detected, 26 variants were confirmed to be germline across seven of the 10 genes included in the panel. Out of the 26 germline variants identified, 20 (76.9%) were pathogenic variants and six (23.1%) were VUS. In 69 of the 95 (73.7%) patients included in the study, no germline variants among the 10 genes tested were identified. Pathogenic variants were found in 20 patients (21.1%): seven in *BRCA1*, six in *BRCA2,* four in *RAD51D*, two in *RAD51C*, and one in *MSH6*. Germline VUS were identified in six patients (6.3%) in *BRCA2*, *MSH6*, *TP53*, and *BRIP1* (Figure 1 and Table 1).

#### 2.2.1. *BRCA1* and *BRCA2* Germline Variants

Of all patients included in this study, 91 (95.8%) were previously tested for *BRCA1* and *BRCA2* variants during clinical testing with BRCA Tumor MASTR^TM^ Plus Dx, and 50 (52.1%) were part of the cohort of a previously published study [16]. Of these 91 patients, 15 had *BRCA1*/*BRCA2* germline variants, and all these variants were detected in this study using the custom panel (Table S1). Among the 95 OC patients included in this study, we identified 15 germline variants in *BRCA1*/*BRCA2* (seven in *BRCA1* and eight in *BRCA2*). All seven variants found in *BRCA1* were pathogenic, five frameshift, one nonsense, and one missense variant. Of the eight variants found in *BRCA2*, three were frameshift, one was a splice site variant at the canonical site -1, and four were missense variants. Of the four missense variants found in *BRCA2*, two were classified as pathogenic, and two were VUS (c.8036A>G and c.9364G>C). The *BRCA2* germline variant c.8036A>G, p.(Asp2679Gly), was identified in patient OC36, diagnosed with HGSOC at 56 years. This variant is a missense VUS predicted to have a deleterious impact on protein by in silico tools and is considered as likely pathogenic in Varsome. However, there is no family history of either breast or OC. Furthermore, this variant was included in a study aimed to assess the clinical relevance of *BRCA2* VUS missense variants and was classified as benign [17]. The variant *BRCA2* c.9364G>C, p.(Ala3122Pro), was identified in patient OC50, diagnosed with HGSOC at 62 years. This variant is predicted to have a deleterious effect on protein by in silico tools and is classified as likely pathogenic in Varsome. In the patient’s family there are four relatives who have been affected with cancer, namely breast, prostate, and colorectal cancer. However, this variant was classified as likely benign in a recent study aimed to classify missense VUS variants in *BRCA1* and *BRCA2* using a multifactorial likelihood model [18].

#### 2.2.2. *RAD51C* and *RAD51D* Germline Variants

**Figure 2:**
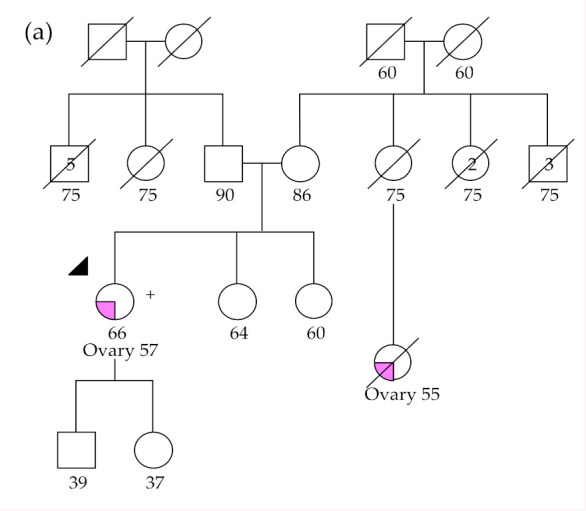

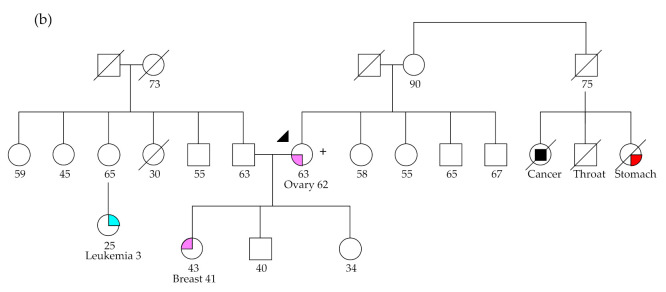
Pedigrees of patients with the *RAD51C* c.709C>T germline variant: (**a**) OC16 and (**b**) OC31. The index patients are indicated by an arrow.

The nonsense variant c.709C>T, p.(Arg327Ter), was detected in *RAD51C* in a proband diagnosed with HGSOC at age of 57 (OC16), who had a first cousin diagnosed with OC (Figure 2a). This variant was also identified in patient OC31, whose family has a history of other cancer types, such as breast, gastric, throat, and leukemia (Figure 2b).

**Figure 3:**
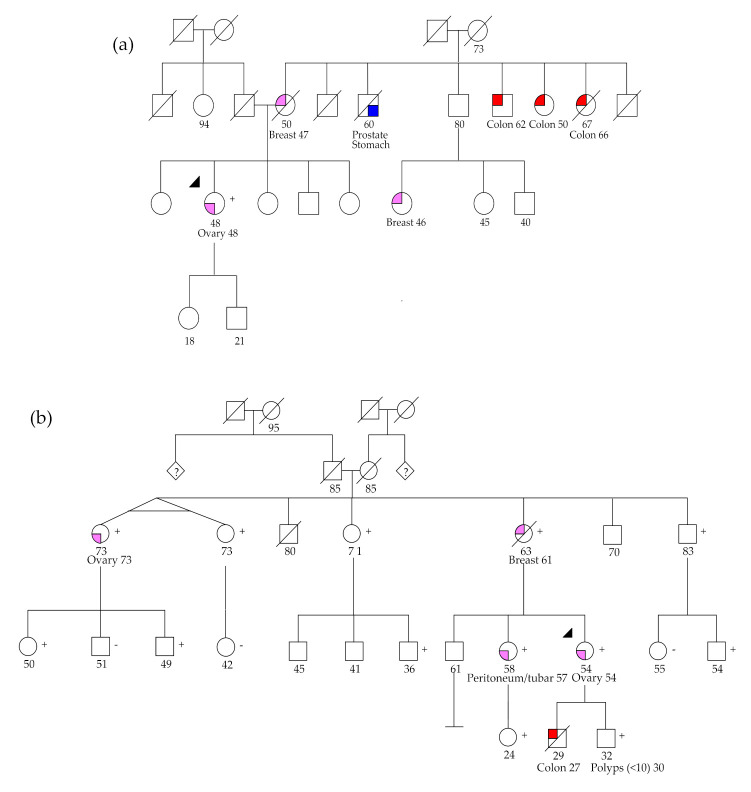
Pedigrees of patients with *RAD51D* c.748del germline variant. Family of patient OC54 (**a**) and OC17, OC30, and OC55 (**b**). The index patient is indicated by an arrow. Plus (+) and minus (−) signals indicate a positive and a negative result for germline genetic testing, respectively.

The *RAD51D* frameshift variant c. 748del, p.(His250ThrfsTer2), was found in patient OC54 with a family history of colorectal, breast, prostate, and gastric cancers (Figure 3a). This variant was also detected in three patients (OC17, OC30, and OC55) belonging to the same family (seemingly unrelated to the previous), in which there is a history of breast and colorectal cancer (Figure 3b).

#### 2.2.3. *MSH6* Germline Variants

**Figure 4:**
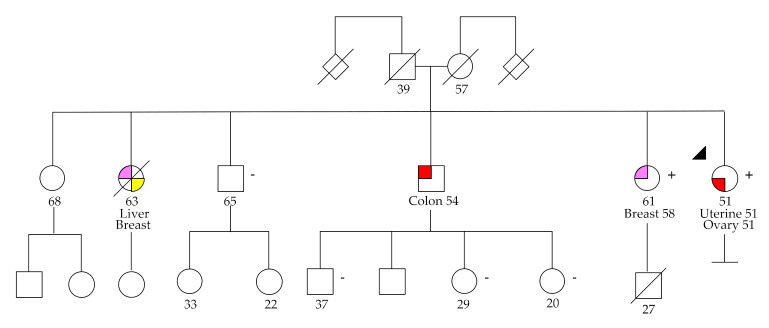
Pedigree of patient OC24 with the likely pathogenic *MSH6* c.3848_3862del germline variant. The index patient is indicated by an arrow.

The *MSH6* inframe variant c.3848_3862del, p.(Ile1283_Tyr1287del), was identified in patient OC24, a woman diagnosed with endometrioid OC and uterine cancer at 51 years old, whose family has a history of colorectal, breast, and liver cancer (Figure 4). There is no classification for this variant in ClinVar, but it is described as a causal variant in the UMD database (www.umd.be). Immunohistochemistry analysis was performed in the ovarian tumor of the patient and showed loss of MSH6 expression. The somatic variant *MSH6* c.4001+1G>T was also identified in this patient, presumably representing the second-hit leading to biallelic inactivation typical of most cancer predisposition tumor suppressor genes.

Two *MSH6* missense VUS were detected in two patients. The *MSH6* c.1729C>T, p.(Arg577Cys), was identified in patient OC37 diagnosed with an ovarian carcinosarcoma at age 68. This variant was reported in ClinVar and in Varsome as uncertain significance, but it is predicted to be damaging by in silico tools. Immunohistochemistry and microsatellite instability analysis were performed in the tumor of the patient, and normal MSH6 expression and no microsatellite instability were observed, thus reducing the likelihood of the variant being associated with an increased risk for OC. The *MSH6* c.3182T>C, p.(Leu1061Pro) variant, was found in patient OC79, who was diagnosed with endometrioid OC at 49 years old and has two relatives affected by colorectal and skin cancer. In ClinVar, there is no classification for this variant, while in VarSome, it is classified as uncertain significance. However, immunohistochemistry analysis performed in the tumor of the proband showed loss of expression for MSH6, suggesting that this variant has a deleterious effect on the protein. Furthermore, we also identified in the OC the somatic variant *MSH6* c.1100A>G, p.(His367Arg), which is classified as pathogenic in ClinVar (reviewed by an expert panel) and could represent the second-hit leading to biallelic inactivation, but the scarce available data currently do not warrant the classification of the germline variant *MSH6* c.3182T>C other than a VUS.

#### 2.2.4. *BRIP1* Germline Variant

The *BRIP1* missense variant c.2477A>G, p.(Asn826Ser), was identified in patient OC11, diagnosed with HGSOC at the age of 39 years and family history of colon cancer and lymphoma. This variant is classified as VUS in ClinVar and in Varsome. However, it is predicted to be damaging by in silico tools

#### 2.2.5. *TP53* Germline Variant

The *TP53* missense variant c.845G>A, p.(Arg282Gln), was identified in patient OC91, diagnosed with pancreatic and thyroid cancer in addition to OC at age of 48. There is history of other cancers in the patient’s family, such as stomach, colorectal, and bone cancer. This variant has conflicting interpretations of pathogenicity in ClinVar (five uncertain significance and two likely pathogenic classifications). However, it was predicted to be damaging by in silico tools and is classified as likely pathogenic in VarSome.

## 3. Discussion

In this study, we aimed to evaluate the yield of germline variants using an NGS customized multi-gene panel for the analysis of ovarian carcinomas. Among the 95 OC patients analyzed, we found that 26 (27.4%) were carriers of rare germline variants in seven genes associated with genetic predisposition to OC (*BRCA1*, *BRCA2*, *BRIP1*, *MSH6*, *RAD51C*, *RAD51D*, and *TP53*). Of these 26 patients, 20 (77%) had pathogenic variants and six (23%) had missense VUS predicted to have a deleterious impact on the protein by in silico tools. Almost all patients included in this study were also tested for *BRCA1* and *BRCA2* somatic and germline variants using a clinically validated method [16], and this study was able to identify all 15 patients known to be carriers of deleterious *BRCA1/BRCA2* germline variants, which allowed validation of the custom panel we designed for those genes. The frequency of pathogenic *BRCA1* and *BRCA2* germline variants in this unselected series of OC patients was 13.7%, which is in concordance with previous studies that reported frequencies between 13% and 18% [12,16,19,20,21]. Furthermore, *BRCA1*/*BRCA2* tumor sequencing allows the detection of both germline and somatic variants in a single test, the latter having a frequency ranging from 4% to 7% [16,22,23]. Thus, about 20% of OC patients are likely to harbor germline or somatic *BRCA1*/*BRCA2* deleterious variants, which are predictive of response to PARPi. To initiate genetic testing of OC patients using a FFPE tumor sample is a strategy that has the potential to significant decrease time and sequencing cost [11], as any clinically actionable variant detected in the initial NGS test can subsequently be evaluated by Sanger sequencing in a peripheral blood sample for its eventual constitutional origin after genetic counseling.

RAD51C and RAD51D are implicated in HR repair of DNA double-strand breaks, playing a key role in replication fork maintenance [24]. Germline variants in *RAD51C* and *RAD51D* are known to confer susceptibility to OC [25,26]. Studies on the prevalence of germline variants in these genes in OC vary widely, ranging between 0.57% and 2.5% for *RAD51C* and 0.16% and 0.57% for *RAD51D*, among patients unselected for age and family history [12,14,27,28,29]. For OC patients selected for family history, the prevalence of germline variants was found to be 1.27% and 0.88% for *RAD51C* and *RAD51D*, respectively [25,26]. The estimated relative risk for OC in carriers of germline variants in the *RAD51C* range from 5.88 to 7.55 [30,31] and from 6.30 to 7.60 for *RAD51D* germline variant carriers [26,31]. The National Comprehensive Cancer Network (NCCN) guidelines for breast and OC recommend that risk-reducing salpingo-oophorectomy (RRSO) should be considered beginning at age 40 to 50 years in women carriers of germline variants in these genes [32]. In this study, we identified two patients (2.1%) with a pathogenic variant in *RAD51C* and four patients (4.2%) with a pathogenic germline variant in *RAD51D*, together representing 30% of all pathogenic germline variants identified. The prevalence of germline variants in *RAD51C* in our cohort (2.1%) is in concordance with previous studies [12,28], while the observed frequency for *RAD51D* (4.2%) is higher than previously reported [26,29]. However, three of the patients with the germline variant in *RAD51D* turned out to be relatives, which may have spuriously increased the contribution of this gene in our study. Genetic counseling has already been offered to healthy relatives of this family, which so far allowed us to identify four female carriers of the germline variant, one of them already submitted to RRSO.

Germline variants in MMR genes (*MLH1*, *MSH2*, *MSH6*, and *PMS2*) are associated with Lynch Syndrome (LS) [33], which is characterized by predisposition to a spectrum of cancers such as stomach, ovary, pancreas, upper urologic tract, and brain tumors, in addition to the most prevalent colorectal and endometrial cancers [34]. A cumulative incidence of 13% at 75 years was observed for OC in carriers of *MSH6* germline variants [35], and the lifetime time risk was estimated to be up to 13.5% by age 70 [36,37]. We identified in one patient with both endometrial and ovarian cancer a *MSH6* germline inframe deletion, which is not described in the literature at the time of writing. Although this variant is predicted to lead to an inframe deletion of five aminoacids at the protein level, we observed loss of MSH6 expression in the tumor by immunohistochemistry and detected a deleterious somatic *MSH6* mutation, presumably representing the second-hit leading to biallelic inactivation. Furthermore, this germline variant has been previously identified in our laboratory in another six index patients presenting clinical criteria for Lynch Syndrome, who also showed consistent MSH6 loss of expression in their tumors, allowing the classification of this variant as likely pathogenic. Because of this genetic diagnosis, the index patient was offered screening for additional tumors associated with Lynch syndrome. Furthermore, genetic counseling and presymptomatic genetic testing were offered to relatives, and carriers will be offered cancer surveillance recommended for Lynch syndrome, with consideration of bilateral salpingo-oophorectomy and hysterectomy after completed childbearing.

One of the side-effects of using larger NGS gene panels is the increased likelihood of finding VUS. On the other hand, it is important to report all additional information to improve variant classification, as some of the VUS may in fact be pathogenic and others may be benign, but with insufficient data to allow their classification either way. As shown in the Results section, we combined in silico, additional somatic mutation, protein expression, and segregation data to classify several germline missense variants, but six of them must remain classified as VUS with the information currently available. Of the two *BRCA2* VUS and two *MSH6* VUS we report here, the evidence favors the pathogenicity of one *MSH6* variant and the benign nature of the remaining three variants. The remaining two VUS were found in other cancer predisposing genes, namely *BRIP1* and *TP53*. BRIP1 binds directly to the BRCT repeats of BRCA1, having an important role in the repair mechanism of double-strand DNA breaks [38]. Deleterious *BRIP1* germline variants have been described as conferring a moderately increased risk for OC [39], with an estimated relative risk of 3.41 [40], showing frequencies ranging between 0.9% and 1.36% [12,14,40]. Finally, *TP53* germline variants cause Li-Fraumeni syndrome, a relatively heterogeneous cancer predisposition syndrome characterized by increased risk of four core cancers (sarcoma, breast carcinoma, brain tumor, and adrenocortical carcinoma) [41], but known to increase the risk for other tumors, including OC [42]. The data we present here on these six VUS may contribute to improving their classification in the future and to clarify their eventual relevance for OC inherited predisposition.

The strategy we propose here using a 10 gene panel for routine OC NGS genetic testing on FFPE samples allowed us to identify 7.4% patients with pathogenic germline variants in genes other than *BRCA1* and *BRCA2*, namely *RAD51C*, *RAD51D*, and *MSH6*, significantly improving the yield of deleterious variants in OC predisposition genes to 21% of the patients tested, without increasing the sequencing costs. Besides the identification of women with inherited cancer predisposition, the use of the NGS multi-gene panel is also useful for the detection of pathogenic variants that are predictive biomarkers for targeted therapies. HR deficient tumors due to deleterious variants in genes other than *BRCA1* and *BRCA2* may be sensitive to PARPi [43,44,45]. In fact, one study showed that RAD51C-deficient cancer cells were highly sensitivity to Olaparib, significantly inhibiting tumor growth in a xenograft model [43], whereas Loveday and colleagues [26] showed that cells deficient in RAD51D were sensitive to Olaparib in a magnitude similar to the observed by silencing *BRCA2*. Furthermore, the Food and Drug Administration (FDA) recently approved Olaparib for the treatment of metastatic castration-resistant prostate cancer (mCRPC) patients with pathogenic germline or somatic variants in HR repair genes, including *RAD51C* and *RAD51D* [46]. However, the evidence that *RAD51C* and *RAD51D* variants are good predictive biomarkers of response to PARPi is still scarce. On the other hand, tumors with MMR deficiency display a phenotype of microsatellite instability, which is a predictive biomarker for immunotherapy [47], allowing the FDA approval of the use of pembrolizumab, an anti-PD1 therapy, for the treatment of solid tumors with high microsatellite instability (MSI-H) or MMR deficiency [48]. The use of multi-gene panel testing that allows the simultaneous detection of both somatic and germline variants in FFPE tumor samples may also be beneficial for the selection of patients to be allocated to clinical trials or for the use of off-label therapy, especially in those who experienced several disease relapses and have limited therapeutic options. Furthermore, since this panel has a customizable design, new genes can easily be incorporated when new clinically actionable information becomes available.

## 4. Materials and Methods

### 4.1. Patients and Samples

The study cohort consisted of 96 women diagnosed with OC at the Portuguese Oncology Institute of Porto (IPO-Porto) from June 2016 to July 2019. The study was approved by the institutional review board (CI-IPOP-35-2016), and written informed consent was obtained from all patients. A FFPE tumor sample was obtained from each patient, after evaluation by a pathologist, who delimited areas with >50% cancer cells. Of all patients included in this study, 91 (95.8%) were previously tested in the clinical routine for *BRCA1* and *BRCA2* variants in tumor and blood using CE-IVD marked methodologies specific for those genes, and 50 patients (52.1%) were part of a study from our group that aimed to test the frequency of germline and somatic variants in a consecutive series of non-mucinous OC patients [16]. The clinicopathological features of OC patients included in the study are presented in Table 2. Patients were diagnosed with OC between 27 and 80 years (median 57 years of age), mostly at advanced stage (78.1% stage III-IV). All patients included in the study were recruited regardless of family history. However, three patients (OC17, OC30, and OC55) recruited at different times were known to belong to the same family when analyzing the results.

### 4.2. DNA Extraction from FFPE Tumor Samples

DNA extraction from FFPE samples was performed using the Cobas^®^ DNA Sample Preparation Kit (Roche Diagnostics, Basel, Switzerland) according to the manufacturer’s protocol. Quality of DNA samples was measured with a Qubit^®^ Fluorometer with the Qubit dsDNA HS assay kit (Thermo Fisher Scientific, Waltham, MA, USA).

### 4.3. Next-Generation Sequencing and Bioinformatic Analyses

NGS was performed in 96 tumor samples using a customized QIASeq Targeted DNA Panel containing ten genes (*BRCA1*, *BRCA2*, *BRIP1*, *MLH1*, *MSH2*, *MSH6*, *PMS2, RAD51C*, *RAD51D*, and *TP53*) (QIAGEN, Antwerp, Belgium). These genes were selected according to international guidelines for OC screening and/or previously description in the literature as conferring a risk for the development of OC. Library preparation was performed according to the manufacturer’s instructions, and final libraries were quantified on a 4200 TapeStation System (Agilent Technologies Inc, Santa Clara, CA, USA). Sequencing was carried out using a high-output kit in the NextSeq 550 System (Illumina, Inc., San Diego, CA, USA) in a 2 × 151 bp paired-end run. Sequencing alignment and variant calling were performed using smCounter2 (QIAGEN, Antwerp, Belgium) [49], and the resulting .vcf files were imported to GeneticistAssistant^TM^ software (SoftGenetics, LLC, State College, PA, USA) for variant annotation. All variants with minor allele frequency (MAF) greater than 1% and synonymous variants were excluded. For MAF filtering, data were obtained from the 1000 Genomes Project (1000G), Genome Aggregation Database (gnomAD), and Exome Aggregation Consortium (ExAC) databases. To screen germline variants, we used the variant allele frequency (VAF) as recommend by ASCO/AMP/CAP guidelines [15] using VAF above 30% as the cutoff, which was shown to be reliable in a previous study of our group [16]. In fact, since the present study is focused on testing tumor suppressor genes, which very often show loss of heterozygosity in the tumor, the VAFs of deleterious germline variants are actually expected to be higher than 50%, which is supported by our earlier finding of mean VAFs of 80% and 69% for *BRCA1* and *BRCA2* pathogenic germline variants, respectively, with the lowest VAF being 49% [16].

### 4.4. Variant Classification

All variants remaining after variant filtering that had been described as pathogenic/likely pathogenic in ClinVar or having literature evidence supporting their pathogenicity were retained as deleterious. Nonsense, frameshift, inframe deletion and/or insertion, as well as canonical splice site variants, were also retained since they are considered to have very strong evidence of pathogenicity [15]. The potential pathogenicity of missense VUS was evaluated using MetaLR and MetaSVM, which combine 10 in silico prediction tools (SIFT, PolyPhen-2 HDIV, PolyPhen-2 HVAR, GERP++, MutationTaster, Mutation Assessor, FATHMM, LRT, SiPhy, and PhyloP) and the maximum minor allele frequency (MMAF) from the 1000G project [50]. We also used Combined Annotation-Dependent Depletion (CADD), which is an integrative annotation built from more than 60 genomic features for scoring the deleteriousness of single nucleotide variants (SNVs) and insertion/deletion variants [51]. Missense VUS were retained only if they were predicted to be damaging by MetaLR, MetaSVM, and CADD. The potential clinical significance of germline VUS was also evaluated using the VarSome approach [52], which is a bioinformatic tool for clinical interpretation of variants according to the American College of Medical Genetics and Genomic (ACMG) and the Association for Molecular Pathology (AMP) guidelines. Variants described in ClinVar as benign/likely benign were classified as benign and discarded.

### 4.5. Germline Variants Validation

Variants suspected to be germline were confirmed by Sanger sequencing in peripheral blood samples. DNA was extracted from blood samples using a standard protocol. Sanger sequencing was performed in a 3500 Genetic Analyzer (Applied Biosystems Foster City, CA, USA) using the BigDye^®^ Terminator v3.1 Cycle Sequencing Kit (Applied Biosystems), following the manufacturer’s instructions.

### 4.6. Microsatellite Instability and Immunohistochemical Analysis

Microsatellite instability analysis was performed using the Bethesda panel of markers (BAT25, BAT26, D2S123, D5S346, and D17S250) and according to the 1997 National Cancer Institute Guidelines, as previously described [53,54]. Assessment of MSH6 protein immunoexpression was performed as previously described [55].

## 5. Conclusions

We conclude that the strategy of reflex gene-panel tumor testing enables the identification of clinically actionable germline variants in a significantly higher proportion of OC patients, while at the same time testing for somatic variants in the same genes, which may be valuable information in patients with advanced disease that have run out of approved therapeutic options. Furthermore, this approach increases the chance to make available genetic counseling, presymptomatic genetic testing, and gynecological cancer prophylaxis to female relatives who turn out to be healthy carriers of deleterious germline variants.

## Figures and Tables

**Table 1:** Germline variants identified among 95 ovarian cancer patients with next generation sequencing of a 10-gene panel.

Patient	Gene	HGVS Coding	HGVS Protein	VAF	ClinVar	MetaLR ^1^	MetaSVM ^1^	C-Score ^1^	Varsome	Additional Tests	Personal History (Age ^2^)	Family History
OC09	*BRCA1*	c.2037delinsCC	p.(Lys679AsnfsTer4)	60%	Pathogenic	N/A	N/A	N/A	N/A	N/A	OC (46)	4 × OC, 2 × PaC, 1 × GC
OC11	*BRIP1*	c.2477A>G	p.(Asn826Ser)	74%	VUS	D	D	26.3	VUS	N/A	OC (39)	1 × CRC, 1 × L
OC12	*BRCA1*	c.211A>G	p.(Arg71Gly)	81%	Pathogenic	N/A	N/A	N/A	N/A	N/A	OC (58)	2 × BC, 1 × PC
OC16	*RAD51C*	c.709C>T	p.(Arg237Ter)	81%	Pathogenic	N/A	N/A	N/A	N/A	N/A	OC (57)	1 × OC
OC17 *	*RAD51D*	c.748del	p.(His250ThrfsTer2)	68%	Pathogenic	N/A	N/A	N/A	N/A	N/A	OC (57)	2 × OC, 2 × BC, 1 × CRC
OC19	*BRCA1*	c.3331_3334del	p.(Gln1111AsnfsTer5)	81%	Pathogenic	N/A	N/A	N/A	N/A	N/A	OC (61)	4 × BC, 1 × OC
OC23	*BRCA1*	c.3817C>T	p.(Gln1273Ter)	92%	Pathogenic	N/A	N/A	N/A	N/A	N/A	OC (46)	6 × BC, 1 × CRC
OC24	*MSH6*	c.3848_3862del	p.(Ile1283_Tyr1287del)	45%	N/A	N/A	N/A	N/A	N/A	IHC: loss of expression	OC (51), UC (51)	2 × BC, 1 × CRC,1 × LC
OC29	*BRCA2*	c.5073dup	p.(Trp1692MetfsTer3)	81%	Pathogenic	N/A	N/A	N/A	N/A	N/A	OC (48), BC (48)	3 × BC, 3 × PC, 3 × CRC
OC30 *	*RAD51D*	c.748del	p.(His250ThrfsTer2)	81%	Pathogenic	N/A	N/A	N/A	N/A	N/A	OC (73)	2 × OC, 2 × BC, 1 × CRC
OC31	*RAD51C*	c.709C>T	p.(Arg237Ter)	77%	Pathogenic	N/A	N/A	N/A	N/A	N/A	OC (62)	1 × BC
OC36	*BRCA2*	c.8036A>G	p.(Asp2679Gly)	36%	CIP	D	D	31	LP	N/A	OC (59)	1 × CRC, 1 ×
OC37	*MSH6*	c.1729C>T	p.(Arg577Cys)	43%	VUS	D	D	34	VUS	IHC: normal; MSI: normal	OC (68)	1 × LC
OC39	*BRCA1*	c.3331_3334del	p.(Gln1111AsnfsTer5)	69%	Pathogenic	N/A	N/A	N/A	N/A	N/A	OC (63)	2 × BC, 1 × CRC
OC40	*BRCA1*	c.3331_3334del	p.(Gln1111AsnfsTer5)	58%	Pathogenic	N/A	N/A	N/A	N/A	N/A	BC (49), OC (50)	---------
OC50	*BRCA2*	c.9364G>C	p.(Ala3122Pro)	88%	VUS	D	D	31	LP	N/A	OC (62)	1 × CRC, 1 × BC, 1 × PC
OC52	*BRCA1*	c.2037_2038insC	p.(Lys680GlnfsTer3)	54%	Pathogenic	N/A	N/A	N/A	N/A	N/A	OC (52)	2 × BC, 1 × PC
OC54	*RAD51D*	c.748del	p.(His250ThrfsTer2)	93%	Pathogenic	N/A	N/A	N/A	N/A	N/A	OC (48)	2 × BC, 1 × PC, 3 × CRC
OC55 *	*RAD51D*	c.748del	p.(His250ThrfsTer2)	56%	Pathogenic	N/A	N/A	N/A	N/A	N/A	OC (54)	2 × OC, 2 × BC, 1 × CRC
OC60	*BRCA2*	c.7975A>G	p.(Arg2659Gly)	62%	Pathogenic	N/A	N/A	N/A	N/A	N/A	OC (56)	3 × BC, 1 × PaC, 1 × CRC
OC72	*BRCA2*	c.5073dup	p.(Trp1692MetfsTer3)	56%	Pathogenic	N/A	N/A	N/A	N/A	N/A	OC (60)	1 × PaC, 1 × PC
OC73	*BRCA2*	c.5073dup	p.(Trp1692MetfsTer3)	86%	Pathogenic	N/A	N/A	N/A	N/A	N/A	OC (44)	2 × BC, 1 × CRC
OC79	*MSH6*	c.3182T>C	p.(Leu1061Pro)	48%	VUS	D	D	24.8	VUS	IHC: loss of expression	OC (49)	1 × CRC
OC88	*BRCA2*	c.2T>G	p.Met1?	93%	Pathogenic	N/A	N/A	N/A	N/A	N/A	BC (59), OC (69)	1 × BC, 1 × PC
OC91	*TP53*	c.845G>A	p.(Arg282Gln)	47%	VUS	D	D	35	LP	N/A	OC (58), TC (60), PaC (60)	1 × GC, 1 × CRC, BC
OC93	*BRCA2*	c.8488-1G>A	---------	57%	Pathogenic	N/A	N/A	N/A	N/A	N/A	OC (65)	1 × BC, 1 × PaC

BC: Breast cancer; CIP: conflicting interpretations of pathogenicity; CRC: Colorectal cancer; GC: gastric cancer; IHC: Immunohistochemistry; L: Lymphoma; LC: Lung cancer; LP: Likely pathogenic; MSI: Microsatellite instability; N/A: not available; OC: ovarian cancer; PaC: Pancreatic cancer; PC: Prostate cancer; UC: uterine cancer; VAF: variant allele frequency; VUS: variant of unknown significance. * OC17, OC30, and OC55 belong to the same family. ^1^ Missense variants were retained if they were predicted to be damaging (D) by MetaLR, MetaSVM, and CADD-score (>15). ^2^ Age at diagnosis.

**Table 2:** Clinical features of the 96 patients included in the study.

Clinical Features		N (%)
Age, median (range years)		57 (27–80)
Figo Stage	Figo stage I	14 (14.6)
	Figo stage II	7 (7.3)
	Figo stage III	55 (57.3)
	Figo stage IV	20 (20.8)
Histological subtype	High-grade serous	72 (71.9)
	Low-grade serous	8 (8.3)
	Clear cell	6 (6.3)
	Endometrioid	7 (7.3)
	Mucinous	1 (1.0)
	Mixed	3 (3.1)
	Carcinosarcoma	2 (2.1)

## Supplementary Materials

The following are available online at https://www.mdpi.com/2072-6694/12/10/2834/s1, Table S1: comparison of results obtained by the custom panel and by a clinically validated test regarding the *BRCA1*/*BRCA2* deleterious variants.
